# Inhibition of the Responses to Sex Pheromone of the Fall Armyworm, *Spodoptera frugiperda*

**DOI:** 10.1673/031.013.13401

**Published:** 2013-11-26

**Authors:** Edi A. Malo, Julio C. Rojas, Rafael Gago, Ángel Guerrero

**Affiliations:** 1 Departamento de Entomología Tropical, El Colegio de la Frontera Sur (ECOSUR), Carretera Antiguo Aeropuerto Km 2.5, C.P. 30700, Tapachula, Chiapas, México; 2 Department of Biological Chemistry and Molecular Modeling, Institute of Advanced Chemistry of Catalonia (CSIC), Jordi Girona 18-26, 08034-Barcelona, Spain

**Keywords:** pheromone inhibition, mating disruptant, antagonism, trifluoromethyl ketones

## Abstract

Trifluoromethyl ketones reversibly inhibit pheromone-degrading esterases in insect olfactory tissues, affecting pheromone detection and behavior of moth males. In this work, (Z)-9-tetradecenyl trifluoromethyl ketone (Z9-14:TFMK), a closely-related analogue of the pheromone of the fall armyworm, *Spodoptera frugiperda* (Smith) (Lepidoptera: Noctuidae), was prepared and tested in electroantennogram and field tests as possible inhibitors of the pheromone action. The electroantennogram parameters, amplitude, and the repolarization time of the antennal responses of *S. frugiperda* males were affected by Z9-14:TFMK vapors. Exposure of male antennae to a stream of air passing through 100 ìg of the ketone produced a significant reduction of the amplitude and an increase of 2/3 repolarization time signals to the pheromone. The effect was reversible and dose-dependent. In the field, the analogue significantly decreased the number of males caught when mixed with the pheromone in 10:1 ratio. The results suggest that Z9-14:TFMK is a mating disruptant of *S. frugiperda* and may be a good candidate to consider in future strategies to control this pest.

## Resumen

Las trifluorometilcetonas inhiben reversiblemente las esterasas que degradan las feromonas en el tejido olfativo de los insectos, afectando la detección de las feromonas y el comportamiento de los machos de lepidópteros nocturnos. En este trabajo se preparó la (Z)-9- tetradeceniltrifluorometilcetona (Z9-14:TFMK), un análogo estructuralmente relacionado con la feromona, con la finalidad de evaluar su actividad en electroantenograma y en pruebas de campo como posible inhibidor de la acción de la feromona sexual. Los parámetros electroantenográficos, amplitud y tiempo de repolarización de la respuesta antenal del gusano cogollero, *Spodoptera frugiperda* (Smith) (Lepidoptera: Noctuidae), fueron afectados por vapores de la Z9-14:TFMK. La exposición de antenas de machos a un flujo de aire pasando a través de 100 µg del análogo produjo una reducción significativa de la amplitud y un incremento de 2/3 del tiempo de repolarización de la señal electroantenográfica a la feromona. El efecto fue reversible y dosisdependiente. El análogo redujo significativamente el número de machos capturados en campo cuando se mezcló con la feromona en una proporción de 10:1. Estos resultados sugieren que el análogo es un disruptor del apareamiento del gusano cogollero y puede ser un buen candidato a considerar en futuras estrategias de control de esta plaga.

## Introduction

The fall armyworm, *Spodoptera frugiperda* (Smith) (Lepidoptera: Noctuidae), is a polyphagous species, widely distributed in the tropical and subtropical regions of the Americas (Andrews 1980), and feeds in more than 60 host plants, although it has a marked preference for gramineous plants (Mitchell 1979). There are two strains of *S*. *frugiperda* that occur sympatrically, one feeds predominantly on corn (the corn strain) and the other on rice and various pasture grasses (the rice strain) (Pashley 1996; Prowell et al. 2004).

The *S. frugiperda* sex pheromone has been studied by a number of authors (Mitchell et al. 1985; Tumlinson et al. 1986; Batista-Pereira et al. 2006; Groot et al. 2008). The sex pheromone composition for the North American population was reported as a mixture of (Z)-9- tetradecen-1-yl acetate (Z9-14:Ac), (Z)-7- dodecen-1-yl acetate (Z7-12:Ac), (Z)-9- dodecen-1-yl acetate (Z9-12:Ac), and (Z)-11- hexadecen-1-yl acetate (Z11-16:Ac) in an 81:0.5:0.5:18 ratio respectively (Tumlinson et al. 1986). The Brazilian population of this species contains an additional component, (E)-7-dodecen-1-yl acetate (E7-12: OAc), that has not been found in the other populations (Batista-Pereira et al. 2006). The pheromone composition also differs in the two host strains. Corn strain females produced significantly more of the second most abundant pheromone compound, (Z)-11-hexadecen-1-yl acetate, and significantly less of most other compounds than rice strain females (Groot et al. 2008).

Pheromone perception in moths and other insects is mediated by olfactory receptor neurons that are localized in long sensilla trichodea of the male antennae (Hansson 1995). After adsorption onto the cuticular surface of the antennae (Kanaujia and Kaissling 1985), pheromone molecules diffuse to the inner cuticular face of the sensory hairs through microscopic pores present in the cuticle of the hair shaft. To diffuse the pheromone into the sensillum lymph, the pheromone is bound to the pheromone-binding protein (Vogt and Riddiford 1981) and transported through the aqueous lymph to the receptor in the dendritic membrane of the olfactory receptor neurons (Vogt and Riddiford 1981; Blomquist and Vogt 2003; Xu et al. 2005). After pheromone stimulation, the sensory neuron returns to its original resting potential by different processes, particularly after the enzymatic degradation of the pheromone (Kaissling 2001). The antennal esterases are key enzymes for the rapid catabolism of pheromone esters (particularly acetates in Lepidoptera), maintaining a low stimulus noise level in sensory hairs (Vogt et al. 1985; Prestwich et al. 1986). The use of inhibitors of these enzymes has been proposed as a potential method for pest control (Prestwich et al. 1986; Renou and Guerrero 2000; Plettner 2002). Trifluoromethyl ketones (TFMKs) are known to inhibit a variety of serine esterases and proteases, particularly the antennal esterases present in male olfactory tissues (Vogt et al. 1985; Duran et al. 1993; Quero et al. 2003). The activity of these compounds arises from the unique features of the fluorine atom, which has a very similar atomic volume as hydrogen and a high electronegativity, which induce fluorinated ketones to form stable hydrates in aqueous solution that are able to produce tetrahedral adducts with the active site of the enzyme (Linderman et al. 1988; Rosell et al. 1996). The development of pheromone antagonists is important for understanding the insect olfactory system and also for practical applications (Plettner and Gries 2010). The aims of this study were: (a) to evaluate the effect of (*Z*)-1,1,1- trifluorohexadec-11-en-2-one (Z9-14:TFMK), a closely-related analogue of the *S. frugiperda* pheromone, on electroantennogram (EAG) parameters as amplitude and repolarization time; and (b) to test whether the analogue disrupts the chemical communication system of *S. frugiperda*.

## Materials and Methods

### Insects

Larvae of *S. frugiperda* were collected from maize, *Zea mays* L. (Poales: Poaceae) at El Manzano colony, municipality of Tapachula, Chiapas, Mexico, and brought to the laboratory for rearing on an artificial diet (Rojas et al. 2003). Pupae were sexed, placed in groups of 20–25 in Petri dishes inside glass boxes (30 × 30 × 30 cm), and maintained in a climatic chamber on a 16:8 L:D photoperiod regime at 25 ± 2° C and 60–70% RH until emergence. Adults were collected daily, separated by sex, and fed with 10% sucrose solution until use.

### Chemicals

Anhydrous tetrahydrofuran and ether were prepared by distillation from sodium/benzophenone under Ar. Anhydrous pentane was obtained from Sigma-Aldrich (www.sigmaaldrich.com). Reactions with airor water-sensitive reagents were carried out in dried glassware under Ar. Proton nuclear magnetic resonance (^1^H NMR), carbon nuclear magnetic resonance (^13^C NMR) and fluorine nuclear magnetic resonance (^19^F NMR) spectra were recorded at 400 or 500, 100, and 376.5 MHz, respectively, as CDCl3 solutions. Electron impact mass spectra were obtained on a gas chromatography-mass spectrometry system using helium as the carrier gas. Z9-14:Ac, Z7-12:Ac, and Z11- 16:Ac were purchased from Sigma-Aldrich (Z)-9-tetradecenol (Z9-14:OH) was purchased from Bedoukian (www.bedoukian.com), and their purity, determined with a gas chromatography-flame ionization detector, was > 97%.

### (*Z*)-1-Iodo-9-tetradecene

A mixture of Z9-14:OH (0.50 g, 2.35 mmol), triphenylphosphine (0.74 g, 2.83 mmol), and imidazole (0.19 g, 2.83 mmol) in dry tetrahydrofuran (16 mL) was stirred at 0° C for 20 min. The flask was protected from light, and then iodine (1.00 g, 2.83 mmol) was added in portions. The mixture was stirred at 0° C for 3.5 hr, quenched with a saturated solution of sodium thiosulfate (10 mL), and extracted with hexane (3 × 10 mL). The combined organic layers were washed with brine, dried, and concentrated to leave a residue, which was purified by flash chromatography over silica gel, eluting with hexane to give the expected iodo-derivative (0.52 g, 69%). IR (film): õ 3004, 2957, 2925, 2853, 1463, 1179, 719 cm^-1^. ^1^H NMR (400 MHz, CDCl3) δ 5.37–5.32 (m, 2H), 3.19 (t, *J* = 7.0 Hz, 2H), 2.03-2.00 (m, 4H), 1.82 (qt, *J* = 7.2 Hz, 2H), 1.40-1.29 (m, 14H), 0.90 (t, *J* = 6.6 Hz, 3H) ppm. ^13^C NMR (100 MHz, CDCl3) δ 130.10, 129.93, 33.71, 32.11, 30.65, 29.85, 29.45, 29.32, 28.67, 27.31, 27.07, 22.51, 14.17, 7.50 ppm.

### Z9-14:TFMK

A 0.7 M solution of *t*-BuLi in pentane (2.40 mL, 1.68 mmol) was added to a solution of (*Z*)-1-iodo-9-tetradecene (0.49 g, 1.51 mmol) in dry pentane/ether (3:2, 16 mL) at -78° C. The mixture was stirred for 5 min at -78° C, then ethyl trifluoroacetate (1.08 mL, 9.09 mmol) was added in one portion and stirred 10 min at -78° C and at room temperature for 10 min more. The mixture was concentrated under reduced pressure, and the residue was purified by flash chromatography over silica gel, eluting with hexane to yield the desired trifluoromethyl ketone (0.25 g, 57%). ^1^H NMR (500 MHz, CDCl_3_) δ 5.38-5.31 (m, 2H), 2.70 (t, *J* = 7.3 Hz, 2H), 2.02-2.01 (m, 4H), 1.67 (qt, *J* = 6.8 Hz, 2H), 1.32-1.29 (m, 14H), 0.89 (t, *J* = 6.0 Hz, 3H) ppm. ^13^C NMR (100 MHz, CDCl_3_) δ 191.82 (q, *J* = 69.72 Hz, CO), 130.11, 129.90, 115.72 (q, *J* = 292.34 Hz, CF_3_), 36.51, 32.10, 29.83, 29.38 (2C), 29.30 (2C), 28.87, 27.29, 27.06, 22.50, 14.15 ppm. ^19^F NMR (376.5 MHz, CDCl_3_) δ -79.76 (CF_3_) ppm. MS (EI) m/z (%): 292 (M+, 29), 110 (30), 97 (67), 96 (53), 95 (54), 84 (47), 83 (80), 82 (71), 81 (71), 70 (78), 69 (91), 68 (64), 67 (72), 57 (59), 56 (78), 55 (100)..

### Electroantennography

Antennal responses of *S. frugiperda* males to the sex pheromone mixture (Z9-14:Ac Z7- 12:Ac, and Z11-16:Ac in an 86:0.3:13.7 ratio, respectively) and to the inhibitor were determined in EAG. Briefly, a live male was inserted and restrained into a universal fit pipette tip (Corning, www.corning.com). The reference electrode was inserted into the neck of 4-day-old males, and the recording electrode was connected to the tip of the antenna, from which the last subsegments had been previously excised. Both electrodes were filled with saline solution (Malo et al. 2004a). The signals generated by the antenna were passed through a high-impedance amplifier (NL 1200; Syntech, www.syntech.nl) and displayed on a monitor equipped with signal processing software (NL 1200, version 2.6; Syntech). A stimulus flow controller (CS-05; Syntech) was used to generate stimuli at 1 min intervals. A flow of humidified pure air (0.7 L/min) was continuously passed over the antenna through the main branch of a 10 mm diameter glass tube. Test solutions (1 and 10 ìg/ìL) of pheromone blend and Z9-14:TFMK were prepared in HPLC-grade hexane. For the intrinsic activity of the inhibitor, the antennae were subjected to puffs of air (1 sec, 0.5 L/min) passing through a Pasteur pipette connected to a lateral branch and containing filter paper (0.5 × 3.0 cm, No. 1, Whatman, www.whatman.com) loaded with 10 µg of the sex pheromone or Z9-14:TFMK. As a control, two puffs of hexane were insufflated at the start and the end of the assay. To each insect antenna, one puff of pheromone and inhibitor was made at 2 min intervals, and a minimum of eight male antennae were considered in each assay. For analysis, control depolarizations were subtracted from the test stimuli values.

For the inhibition test, vapors of Z9-14:TFMK were applied to the antennae by turning on a flux of air (0.1 L/min), which passed through a Pasteur pipette containing filter paper with 1, 10, and 100 µg of the inhibitor. Pheromone stimulations were performed as above. Three EAG responses to the pheromone were measured at 2 min intervals in pure air, and the average value was considered as the response before treatment. Then, the Z9-14:TFMK flux was turned on for 2 min, and a series of three depolarizations to the pheromone was recorded (response during treatment). The airflow containing Z9-14:TFMK was turned off, and after 5 min of antennal recovery, three new stimulations with pheromone were performed (response after treatment). Antennae of 11 males (only one antenna of each male) were used. The EAG recordings were stored on a computer, and their maximum amplitude and repolarization time at 2/3 of the baseline (2/3 RT) were determined, as described in Perez- Luis et al. (2010).

### Field tests

The trials were performed in the sorghum field at El Manzano colony. In this area, two crop cycles are grown annually. Sorghum or maize, watered by sprinkler irrigation, are grown from January to May, and soybean is grown during the rainy season, from July to November (Malo et al. 2004b). In the first experiment, the activity of Z9-14:TFMK alone was tested with regard to the pheromone in a fully randomized block design. In each block, four traps per treatment were deployed and four blocks were considered. The numbers of males caught by the four traps within the block were combined on each observation date, and this constituted a replicate. The blocks were arranged in parallel lines about 50 m apart in a sorghum field (45 ha). Traps were placed at a height of 1.5 m above ground and separated by 50 m from one another. Lures consisted of rubber septa containing 100 µg of pheromone (mixture of Z9-14:Ac, Z7-12:Ac, and Z11-16:Ac in an 86: 0.3:13.7 ratio, respectively) (Malo et al. 2001) or 100 µg of Z9-14:TFMK. Scentry *Heliothis* traps (Ecogen, Inc., Billings, MT) were used for the experiments. The traps were placed on 21 March to 9 April 2010, rotated within blocks, and the lures were renewed every 10 days. Trap captures were recorded every three to four days for a total of six observation dates.

In the second experiment, the attractiveness of 1:0.1, 1:1, and 1:10 mixtures of the pheromone (100 µg) and the TFMK (10, 100, and 1000 µg) were tested in similar septa and traps. Traps were deployed in a fully randomized block design with six replicates for each treatment. The numbers of males caught by the six traps within the block were combined on each observation date, and this constituted a replicate. Trap catches were recorded every three days for a total of eight observation dates. The experiment lasted from 14 February to 9 March 2011. On each observation date, traps were emptied and the number of males caught was recorded. Voucher specimens were brought to the insect collection of El Colegio de la Frontera Sur (Tapachula, Chiapas, Mexico).

### Statistical Analysis

All statistical analyses were performed using the computer package Statistica (StatSoft 2003). When necessary, the results were transformed to √x to meet the assumptions of normality and homogeneity of variances. The EAG neat (recordings minus control) mean responses to Z9-14:TFMK in comparison to the pheromone blend were analyzed by the matched-pairs *t*-test. For the inhibition experiments, the differences in EAG amplitude and 2/3 RT before, during, and after treatment were also analyzed by the matched-pairs *t*test. For the field experiments, the data were analyzed as number of moths captured/ trap/night. The results of the Z9- 14:TFMK activity in comparison to the pheromone were analyzed by the matched-pair *t*test, whereas data of inhibition of captures were analyzed by one-way ANOVA followed by a posthoc Tukey test for multiple comparisons of the means (*p* < 0.05). The level of probability considered significant in all analysis was *p* ≤ 0.05.

## Results

### Electroantennogram

Male antennae responded positively in EAG tests to puffs of the pheromone blend and the analogue. In average (± SE), the antennal responses to the pheromone (2.51 ± 0.37 mV) were higher than those to the TFMK (1.10 ± 0.24 mV) (*t* = 4.67; df = 7; *p* < 0.01) (Figure 3), which in turn were higher than the control (hexane) (data not shown).

Exposure of the antenna to air loaded with Z9- 14:TFMK (100 µg) resulted in a significant reduction of the EAG amplitude and an increase of the 2/3 RT to the pheromone responses, the effect being reversible (Figure 4). Thus, the amplitude before the treatment was 2.96 ± 0.16 mV vs. 2.03 ± 0.12 mV during exposure (*t* = 4.6; df = 10; *p* < 0.001) and 2.97 ± 0.19 mV after treatment (Table 1). Similarly, the 2/3 RT of the pheromone response was significantly increased from 704.9 ± 23.5 ms in pure air to 958.4 ± 42.4 ms in the presence of the inhibitor (*t* = 3.1; df = 10; *p* < 0.01) and returned to 725.2 ± 24.8 ms after the experiment.

**Table 1.**
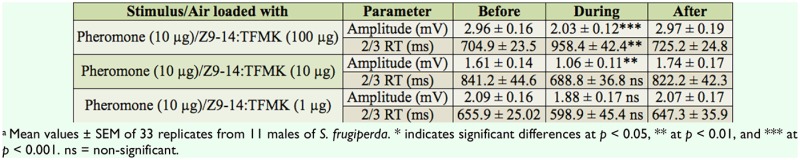
Mean values of the EAG amplitude and 2/3 repolarization time (2/3 RT) of the responses of *Spodoptera frugiperda* male antennae before, during and after exposure to Z9-14:TFMK loaded air.

When the antenna was subjected to Z9- 14:TFMK (10 µg) loaded air, the amplitude to the pheromone response decreased, but the 2/3 RT value was not affected. Thus, whereas the mean amplitude before the treatment was 1.61 ± 0.14 mV, it was 1.06 ± 0.11 mV (*t* = 4.38; df = 10; *p* < 0.01) during exposure and 1.74 ± 0.17 mV after the treatment (Table 1). The 2/3 RT of the pheromone response decreased from 841.2 ± 44.6 ms in pure air to 688.8 ± 36.8 ms in the presence of the analogue (*t* = 3.1; df = 10; *p* > 0.05) and returned to 822.2 ± 42.3 ms after the treatment. The effects of both parameters were again fully reversible (Table 1).

No significant effect on the pheromone response was apparent when 1 µg of Z9- 14:TFMK was tested. Thus, the mean amplitude was 2.09 ± 0.16 mV before the treatment, 1.88 ± 0.17 mV (*t* = -1.99; df = 10; *p* > 0.05) during exposure, and 2.07 ± 0.17 mV after the treatment (Table 1). Similarly, the effect on the 2/3 RT was not significant (655.9 ± 25.02 ms in air vs. 598.9 ± 45.4 ms (t = -1.90; df = 10; *p* > 0.05) in the presence of the analogue, and 647.3 ± ms after the treatment).

### Field Tests

In a previous trial to discover the intrinsic activity of the analogue, traps baited with Z9- 14:TFMK alone caught a significantly lower number of males (0.08 ± 0.1 males/trap/day) than traps baited with the pheromone blend (0.51 ± 0.04 males/trap/day) (*t* = 4.17; df = 23; *p* < 0.001) (data not shown).

In the antagonist assay, the number of males caught with mixtures of pheromone and inhibitor in a 1:10 ratio was significantly reduced when compared to that caught by traps baited with pheromone alone (*F* = 10.91, df = 3, 28; *p* < 0.001) (Figure 5). When traps were baited with the antagonist in a 1:1 ratio, the number of males captured was also lower than that caught by the pheromone alone, but the difference was not significant (Figure 5). In the same regard, no effect was observed when the pheromone was mixed with the antagonist in 1:0.1 ratio (Figure 5).

## Discussion

The enzymatic systems present in male antennae are responsible for degradation of pheromones, thus the inhibition of these enzymes may disrupt the pheromone communication of moths. Previous results agree with this assessment (Bau et al. 1999; Riba et al. 2001, 2005; Solé et al. 2008a), so in this study, (Z)-9-tetradecenyl trifluoromethyl ketone (Z9-14:TFMK), a closely related analogue of the major sex pheromone component of *S. frugiperda*, was prepared and was tested in EAG and field tests as a possible inhibitor of the pheromone action. The results show that Z9-14:TFMK elicited a modest EAG response (56% relative to the pheromone), which is in line with the electrophysiological activity of other ketones that are structurally analogues of the pheromone of other lepidoptera. Liljefors et al. (1984) reported modest EAG activity of (Z)-10-pentadecen-2-one, the corresponding analogue of the pheromone of the turnip moth, *Agrotis segetum*. In the same way, (Z)-16- nonadecen-14-yn-2-one, the analogue resulting from the same replacement on the pheromone structure of the processionary moth, *Thaumetopoea pityocampa*, displayed moderate electrophysiological activity (Parrilla and Guerrero 1994). Z11-14:TFMK, structurally similar to the pheromone of the *Z* strain of the European corn borer, *Ostrinia nubilalis*, also showed limited electrophysiological activity in comparison to the pheromone (37.4 ± 10.6%) (Solé et al. 2008a).

In inhibition tests, Z9-14:TFMK evoked a dose-dependent effect on the EAG kinetics of *S. frugiperda* males. A 100 ìg dose of the chemical resulted in a decrease of the pheromone signal both in amplitude and 2/3RT, but at 10 ìg dose only the amplitude was affected. No effect was observed at the minimum dose of 1 ìg. The dose of the compound, therefore, appears to be critical to affect the EAG pheromone kinetics of *S. frugiperda* males. The effects were fully reversible, with the initial values of both parameters being completely recovered 5 min after treatment. The kinetics of the EAG signals are affected by several factors, such as temperature (Bestmann and Dippold 1989; Kodadova and Kaissling 1996), stimulus concentration (Alcorta, 1991), and type of compound (Rumbo et al. 1995). The results of our study agree with those of Renou et al. (1997), in which a potent esterase inhibitor, 3-octylthio-1,1,1-trifluoropropan-2- one (commonly known as OTFP), was found to decrease the EAG amplitude and increase the RT of the pheromone of *Spodoptera littoralis*. This analogue also modified the EAG responses of *Mamestra brassicae* and *Helicoverpa zea* to their pheromones (Renou et al. 1997). In addition, TFMKs elicited a significant reduction of the single sensillum responses to the pheromone of *S. littoralis* (Renou et al. 1997), *A. polyphemus* (Pophof et al. 2000), and *Sesamia nonagrioides* (Quero et al. 2004). The results of our study point to a possible esterase inhibition and/or competitive binding to the pheromone receptors. In this context, it was shown that some TFMKs bind a PBP present in the sensory hairs of the processionary moth to facilitate transportation of these analogues through the haemolymph, in competition with pheromone molecules, to inhibit catabolic enzymes (Feixas et al. 1995). Also, in binding experiments on antennal extracts of *Mamestra brassicae*, *Z*11*-*16:TFMK was able to displace the major component of the pheromone (Campanacci et al. 1999). Binding of the TFMKs to the PBPs may impair the pheromone scavenging role of these proteins, thus contributing to the EAG recovery delay.

Different mechanisms of action may explain the effects of TFMKs as inhibitors of the sex pheromone. First, they may act on olfactory receptor neurons tuned to the pheromone components. For instance, *Z*11*-*16:TFMK increased the firing activity of the alcohol and aldehyde neurons in *S*. *nonagrioides* male sensilla and decreased the responses of the pheromone (acetate) receptor neurons to the pheromone (Quero et al. 2004). Second, TFMKs can also be bound to the PBP in competition with pheromone molecules (Feixas et al. 1995; Pophof et al. 2000). These effects are consistent with the disruption of male upwind flights induced on *S*. *nonagrioides*, *O*. *nubilalis*, and *S. littoralis* males when attracted to virgin females or pheromone lures in a wind tunnel (Bau et al. 1999; Riba et al. 2001, 2005; Guerrero et al. 2003).

In the field, Z9-14:TFMK behaved as an effective antagonist of the pheromone action when mixed with the natural attractant in a 10:1 ratio, the effect being dose-dependent. Dissimilar results were obtained in 1:1 and 0.1:1 inhibitor:pheromone mixtures, because whereas the former formulation displayed an antagonist effect, the latter was apparently synergist, but in neither case were the results significant. Overall, these results agree with those previously reported on *S. nonagrioides* (Riba et al. 2001), *O. nubilalis* (Riba et al. 2005; Solé et al. 2008a), *Cydia pomonella* (Giner et al. 2009), and *Zeuzera pyrina* (Muñoz et al. 2011), emphasizing the potential utilization of these compounds in pest control. Moreover, the low toxicity displayed by TFMKs has been noticed on Swiss mice (Ashour and Hammock 1987; Riba et al. 2001) and in aquatic toxicity studies on algae growth and survival (Rosa et al. 2006). The low toxicity is probably associated to their reversible mechanism of action, in contrast to other chemicals that are much more toxic irreversible inhibitors of carboxylesterases.

In summary, Z9-14:TFMK elicited a significant reduction of the EAG pheromone responses in *S*. *frugiperda*, and behaved as a pheromone antagonist in the field. The results suggest that this pheromone analogue may be a good candidate to consider as a mating disruptant in future strategies to control *S. frugifrugiperda*. In this context, it is worth noting the notable reduction of damage induced by the maize pests *S. nonagrioides* and *O. nubilalis* upon treatment of infested fields with Z11- 16:TFMK in large scale experiments (Solé et al. 2008b).

**Figure 1.**
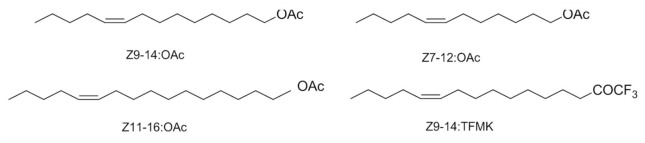
Structures of (*Z*)-9-tetradecenyl acetate (Z9-14:OAc), (Z)-7-dodecenyl acetate (Z7-12:OAc), (Z)-11-hexadecenyl acetate (Z11-16:OAc), pheromone components of *Spodoptera frugiperda*, and (*Z*)-9-tetradecenyl trifluoromethyl ketone (Z9-14:TFMK), the antagonist considered in this work. High quality figures are available online.

**Figure 2.**
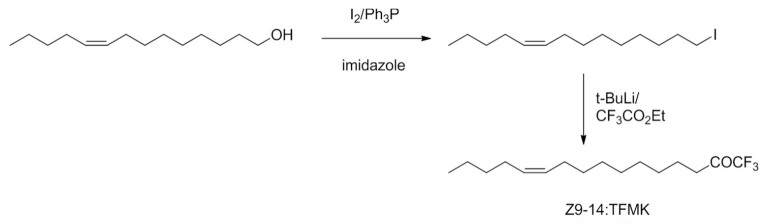
Synthetic approach of Z9-14:TFMK. High quality figures are available online.

**Figure 3.**
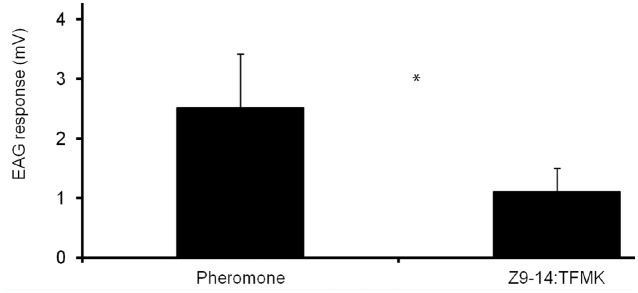
Neat EAG mean responses of a male antenna of *Spodoptera frugiperda* to puffs of 10 µg of the sex pheromone blend and Z9-14:TFMK. * indicate significant differences at *p* < 0.01 (matched-pairs *t*-test). High quality figures are available online.

**Figure 4.**
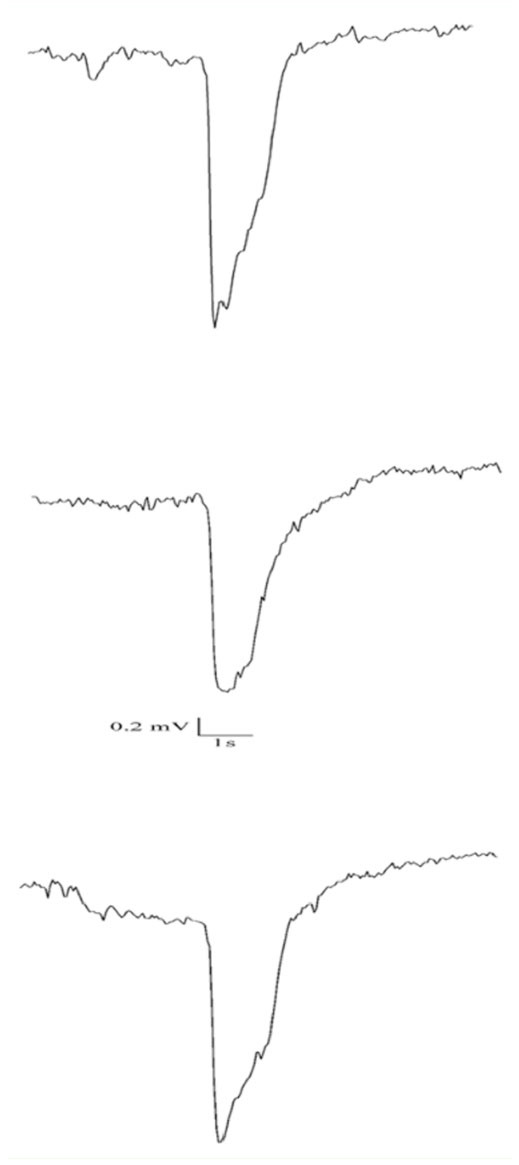
EAG traces of a *Spodoptera frugiperda* male antenna in response to puffs of pheromone (10 µg) before (upper), during (middle), and after (lower) exposure to air loaded with Z9-14:TFMK (100 mg). Vertical scale: bar = 0.2 mV; horizontal scale: bar = 1 s. High quality figures are available online.

**Figure 5.**
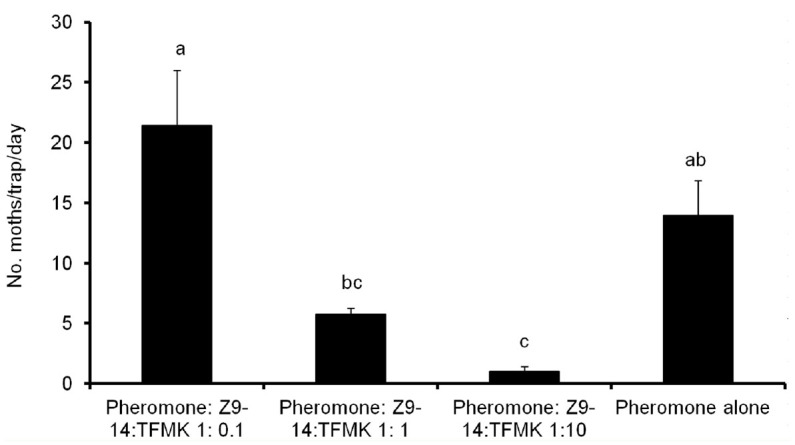
Mean number of *Spodoptera frugiperda* males caught in traps baited with the sex pheromone alone and mixtures of the pheromone and Z9-14:TFMK in 1:0.1, 1:1 and 1:10 ratio. Bars followed by the same letter are not significantly different (One-way ANOVA followed by posthoc Tukey test for multiple comparisons of means (*p* < 0.05)). High quality figures are available online.

## Abbreviations

**2/3 RT**,: repolarization time at 2/3 of the baseline;
**EAG**,: electroantennogram;
**NMR**,: nuclear magnetic resonance;
**TFMK**,: Trifluoromethyl ketone;
**Z7-12:Ac**,: (Z)-7-dodecen-1-yl acetate;
**Z9-12:Ac**,: (Z)-9-dodecen-1-yl acetate;
**Z9-14:Ac**,: (Z)-9- tetradecen-1-yl acetate;
**Z9-14:TFMK**,: (*Z*)-1,1,1-trifluorohexadec-11-en-2-one;
**Z11-16:Ac**,: (Z)-11-hexadecen-1-yl acetate
